# Supplementary material for: TrkB Inhibits the BMP Signaling-Mediated Growth Inhibition of Cancer Cells
Source: Cancers (Basel). 2020 Jul 28;12(8):2095. doi: 10.3390/cancers12082095 (PMC7464134; doi:10.3390/cancers12082095)
Supplement: Supplementary file 1 [file cancers-12-02095-s001.pdf]

Supplementary Figures

TrkB inhibits BMP signaling-mediated growth inhibition of cancer cells

Min Soo Kim, and Wook Jin *

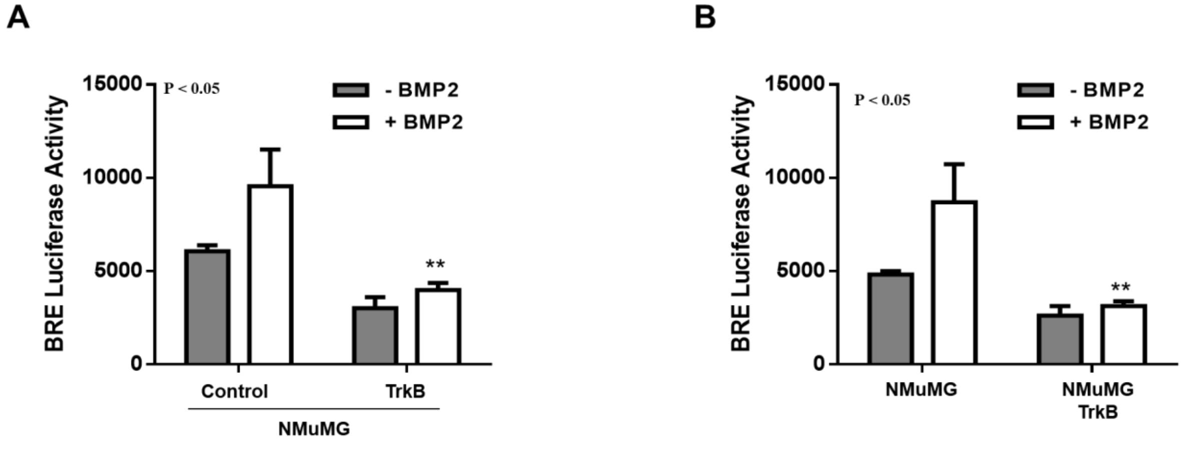

Supplementary Figure 1. TrkB inhibited BMP signaling. (A) BMP-2-responsive BRE Luciferase reporter activity in NMuMG cells transfected with TrkB. **Control versus treatment with BMP-2, $p < 0.05$, n = 3. (B) BMP-2-responsive BRE Luciferase reporter activity in NMuMG and NMuMG-TrkB cells. **Control versus treatment with BMP-2, $p < 0.05$, n = 3.

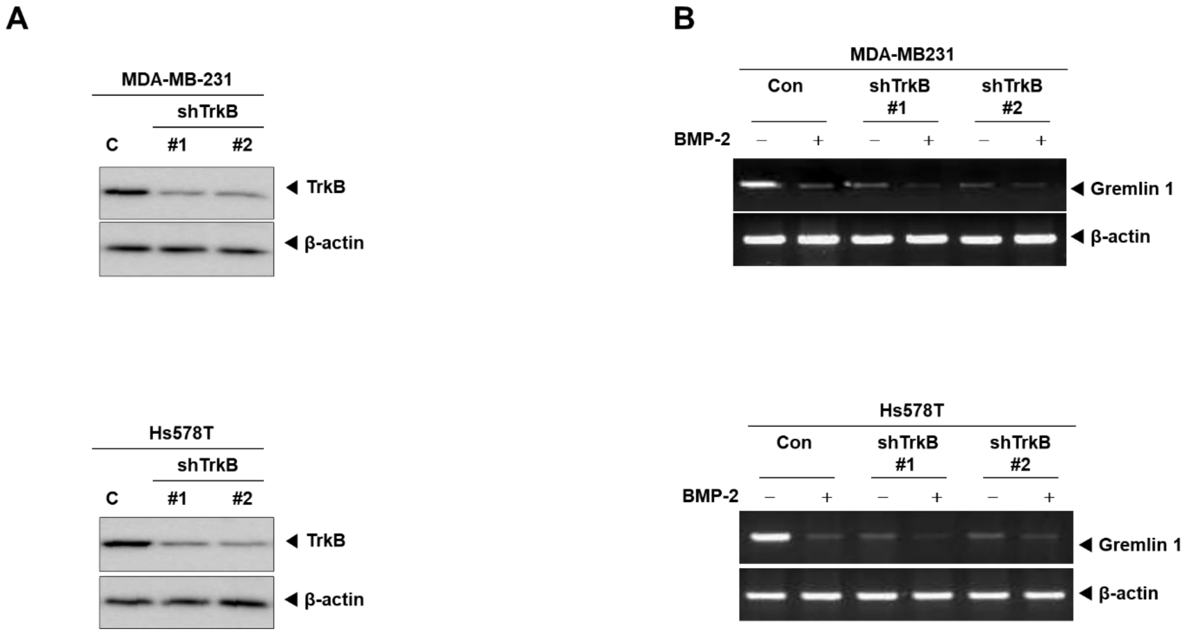

Supplementary Figure 2. TrkB and Gremlin 1 expression in MDA-MB-231 and Js578T cells following shRNA transfection. Western blot analysis of TrkB (A) and RT-PCR analyses of Gremlin 1 mRNA(B) expression in Hs578T and MDA-MB-231 control-shRNA or TrkB-shRNA cells.

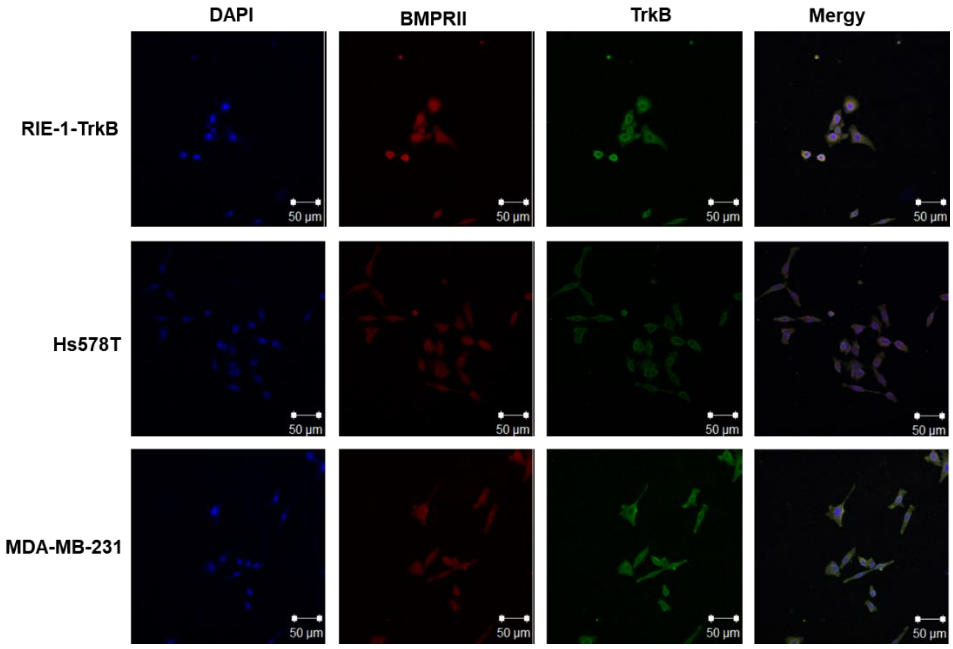

Supplementary Figure 3. The co-localization of TrkB and BMPRII. Immunofluorescence staining of BMPRII and TrkB in Hs587T, MDA-MB-231, and RIE-1-TrkB cells. Scale bar represents 50 μm.

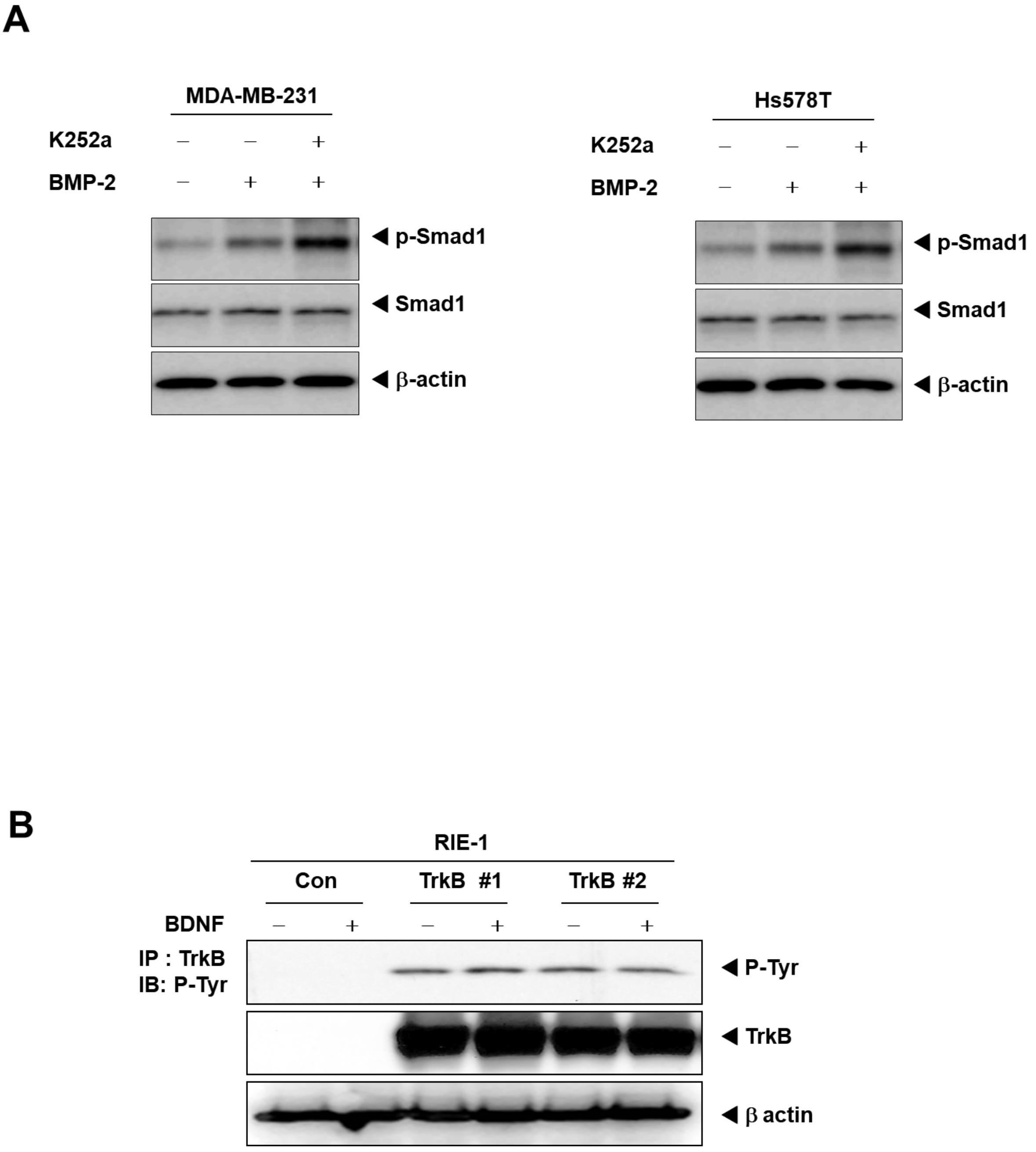

Supplementary Figure 4. Inhibition of TrkB enhanced SMAD1 activation via BMP-2. (A) Western blot analysis of phospho-SMAD1 and SMAD1 expression in Hs578T and MDA-MB-231 cells with or without BMP-2 (5 ng/mL) or K252a (50 nM) treatment. (B) Western blot analysis of TrkB phosphorylation in RIE-1 and RIE-1-TrkB cells.

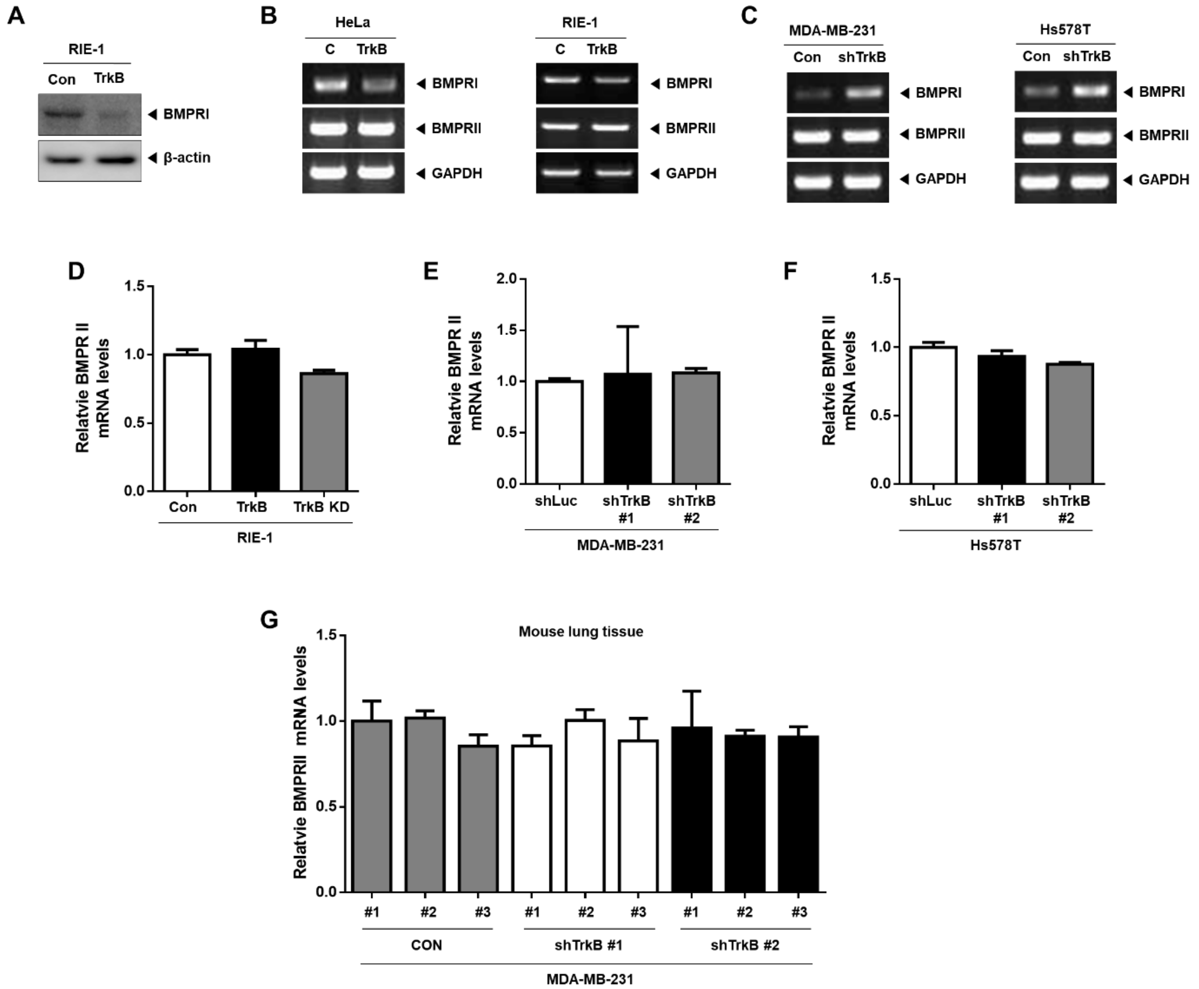

Supplementary Figure 5. TrkB suppressed BMPRI expression. (A) Western blot analysis of BMPRI expression in RIE-1 and RIE-1-TrkB cells. Loading control: β-actin. (B) RT-PCR analyses of BMPRI and BMPRII mRNAs in RIE-1 or HeLa cells with or without TrkB transfection. (C) RT-PCR analyses of BMPRI and BMPRII mRNAs in Hs578T or MDA-MB-231 cells transfected with the control or TrkB shRNA. (D) Relative expression of the BMPRII mRNA in RIE-1, RIE-1-TrkB, and RIE-1-TrkB KD cells, as determined by quantitative RT-PCR. Loading control: 18S. $p < 0.05$, t-test. (E) Relative expression of the BMPRII mRNA in MDA-MB-231 cells transfected with the control or TrkB shRNA, as determined by quantitative RT-PCR. Loading control: 18S. $p < 0.05$, t-test. (F) Relative expression of the BMPRII mRNA in Hs578T cells transfected with the control or TrkB-shRNA, as determined by quantitative RT-PCR. Loading control: 18S. $p < 0.05$, t-test. (G) Relative expression of the BMPRII mRNA in the lungs of mice harboring either MDA-MB-231 control-shRNA or TrkB-shRNA cells, as determined by quantitative RT-PCR. Loading control: 18S. $p < 0.05$, t-test.

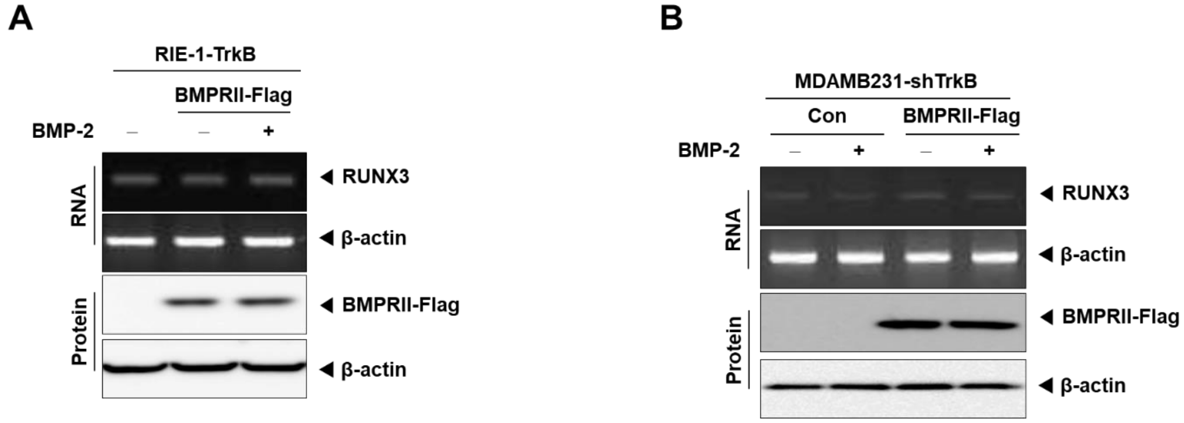

Supplementary Figure 6. TrkB-mediated BMPRII upregulation did not induce RUNX3 expression. (A) Relative expression of the RUNX3 mRNA and protein in RIE-TrkB cells transfected with BMPRII and treated with BMP-2 (5 ng/mL). (B) Relative expression of the RUNX3 mRNA and protein in MDA-MB-231 TrkB-shRNA cells transfected with BMPRII and treated with BMP-2 (5 ng/mL).

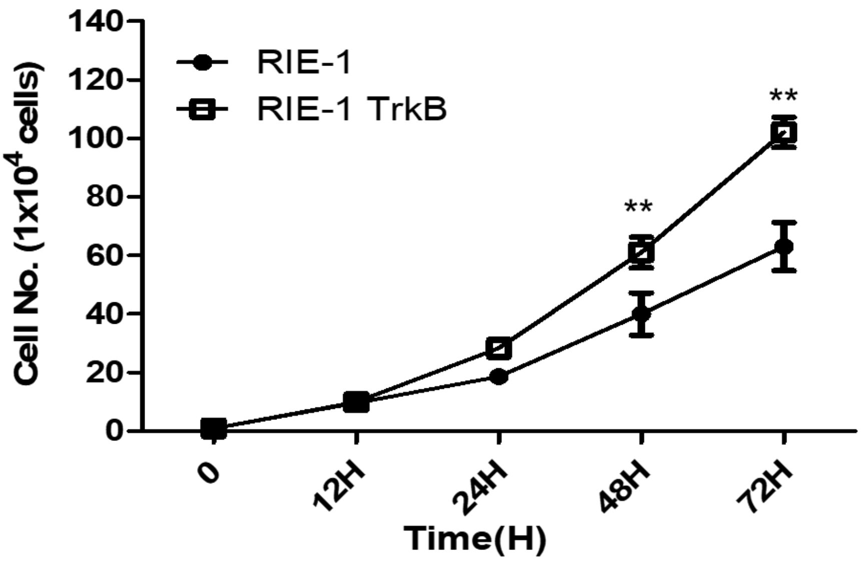

Supplementary Figure 7. Growth of RIE-1 and RIE-1-TrkB cells. Each data point represents the mean number of cells counted in three dishes. **RIE-1 versus RIE-1-TrkB, $p < 0.05$, n = 3.

Supplementary Table 1. Primer sequences for Cloning, RT-PCR

Gene	Primer Sequence
For qRT-PCR	
BMPR I	F: 5′- CAGGTTCCTGGACTCAGCTC -3′<br>R: 5′- CACACCACCTCACGCATATC -3′
BMPR II	F: 5′- AGACTGTTGGGACCAGGATG -3′<br>R: 5′- GTCTGGCCCACTGAATTGTT -3′
18S	F: 5′- TCGGTCTTATTTTGCTGGTTT -3′<br>R: 5′- ATCGCTAGTTGGCATCGTTT -3′
For RT-PCR	
BMPR I	F: 5′- GATGGCTGGTTTTGCTCATT -3′<br>R: 5′- CTCCATATCGGCCTTTACCA -3′
BMPR II	F: 5′- CAGCAGAACCTTCCCAAGAG -3′<br>R: 5′- CTTGGGCCCTATGTGTCACT -3′
RUNX3	F: 5′- TTTCACCCTGACCATCACTG -3′<br>R: 5′- TCGGAGAATGGGTTCAGTTC -3′
Gremlin 1	F: 5′- GCTCTGGCATTCAGAGAACC -3′<br>R: 5′- AAATTCGCCTAGCGTGAGAA -3′
β-actin	F: 5′-TCCCTGGAGAAGAGCTACGA-3′<br>R: 5′-AGCACTGTGTTGGCGTACAG-3′

© 2020 by the authors. Submitted for possible open access publication under the terms and conditions of the Creative Commons Attribution (CC BY) license (http://creativecommons.org/licenses/by/4.0/).

Detailed Information about Western Blot

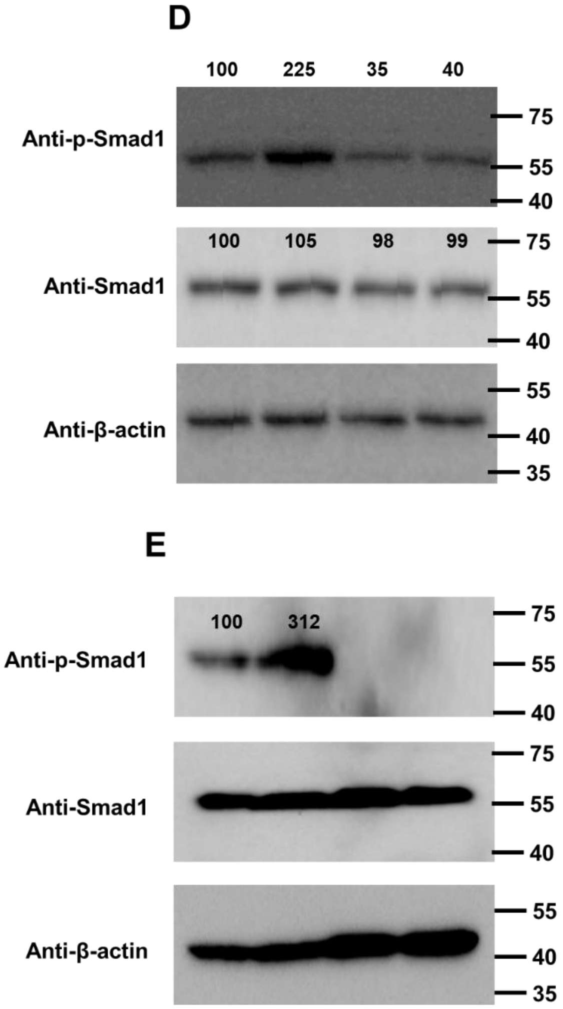

Figure S8. Detailed information about western blot of Figure 1D, E.

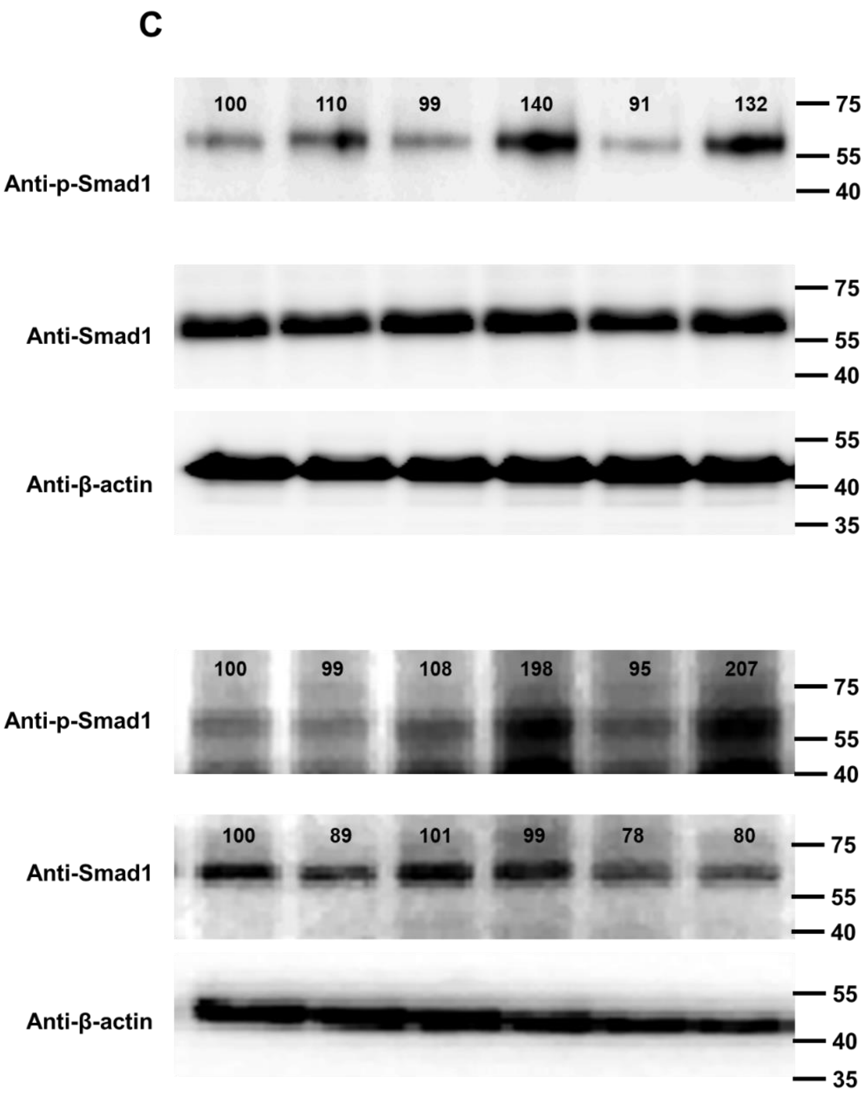

Figure S9. Detailed information about western blot of Figure 2C.

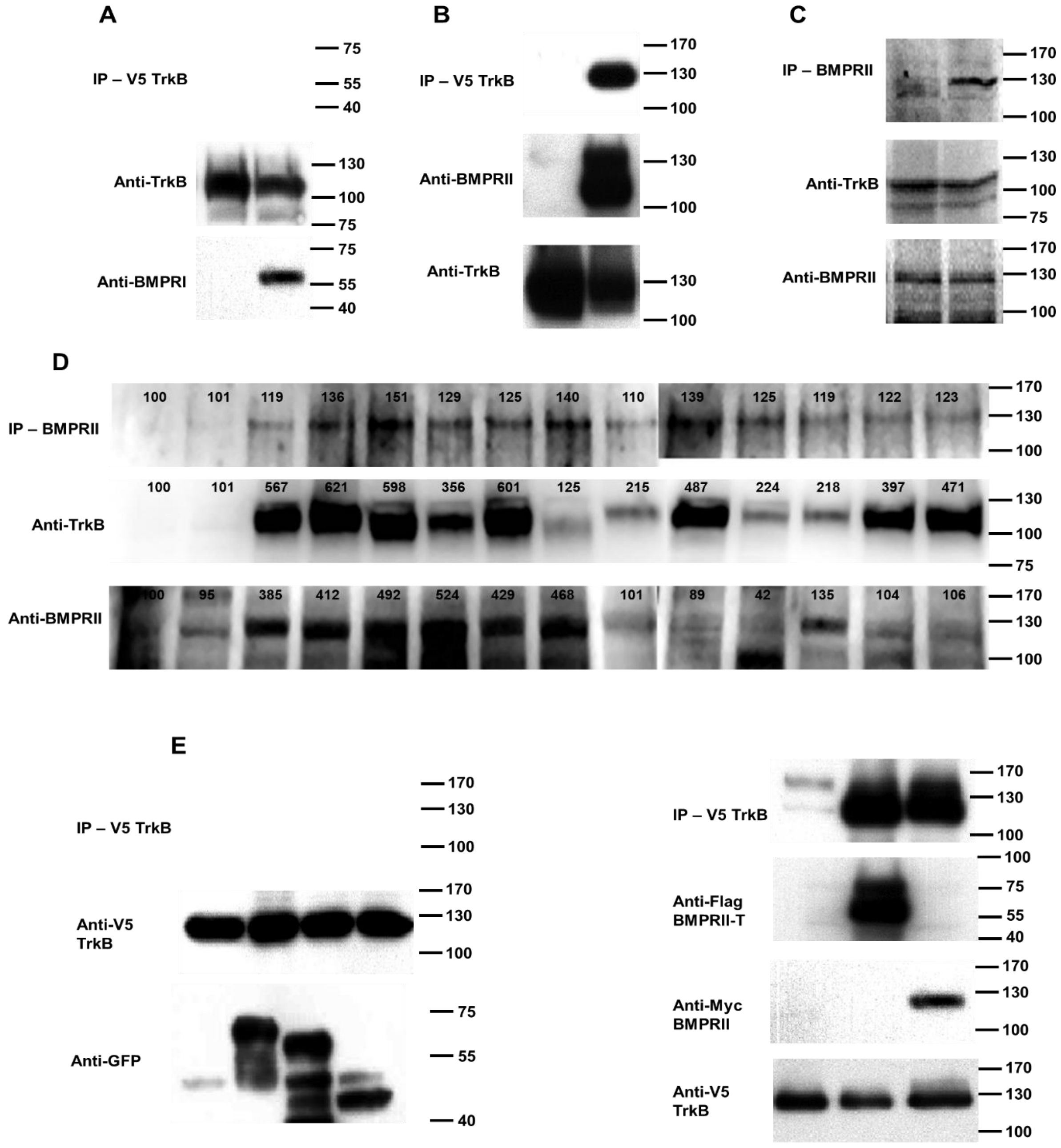

Figure S10. Detailed information about western blot of Figure 3A-C, 3E.

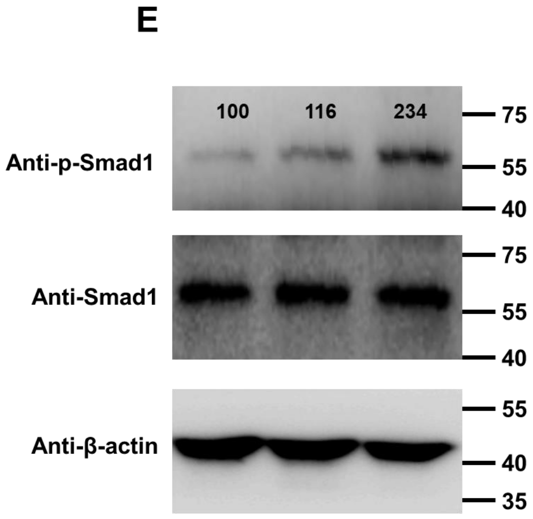

Figure S11. Detailed information about western blot of Figure 4E.

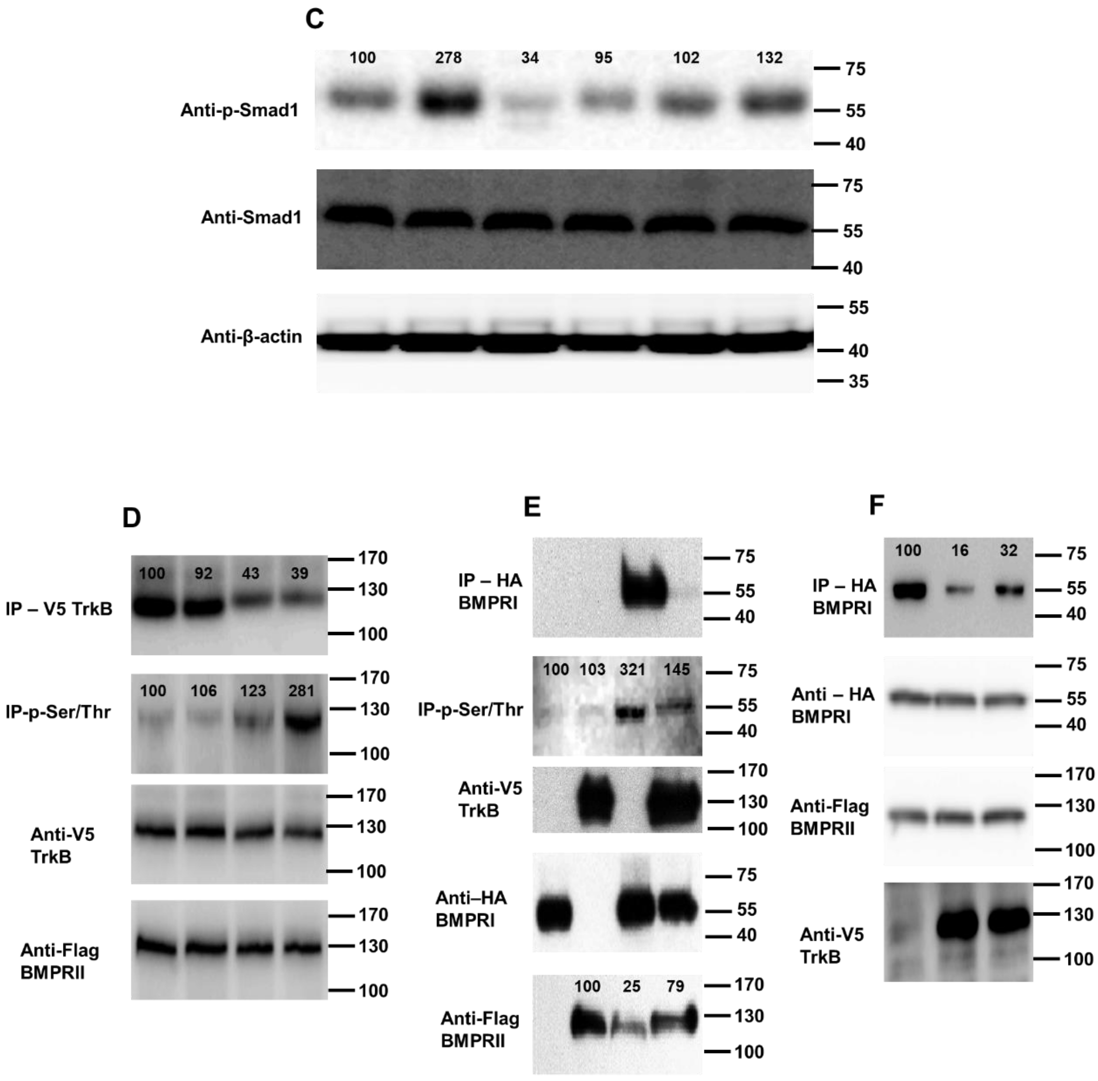

Figure S12. Detailed information about western blot of Figure 5C–F.

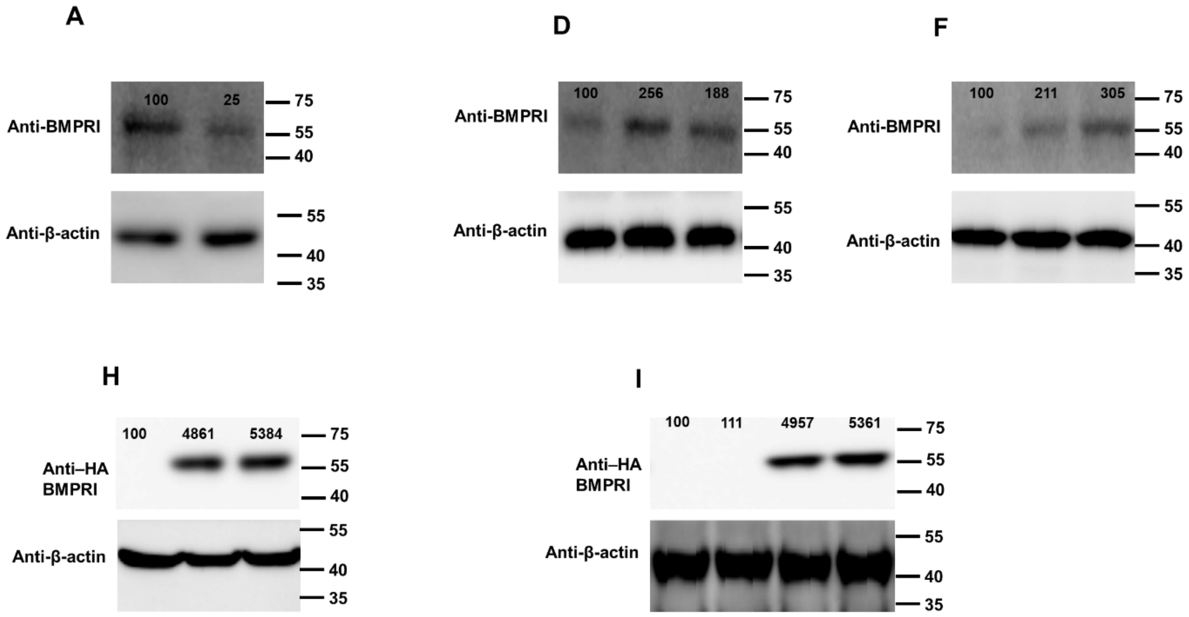

Figure S13. Detailed information about western blot of Figure 6A,D,F,H,I.

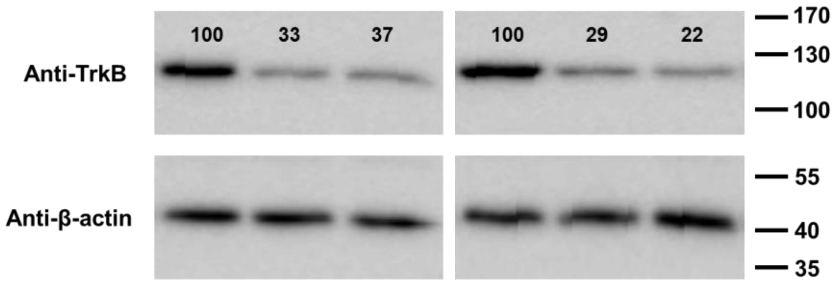

Figure S14. Detailed information about western blot of Figure S2.

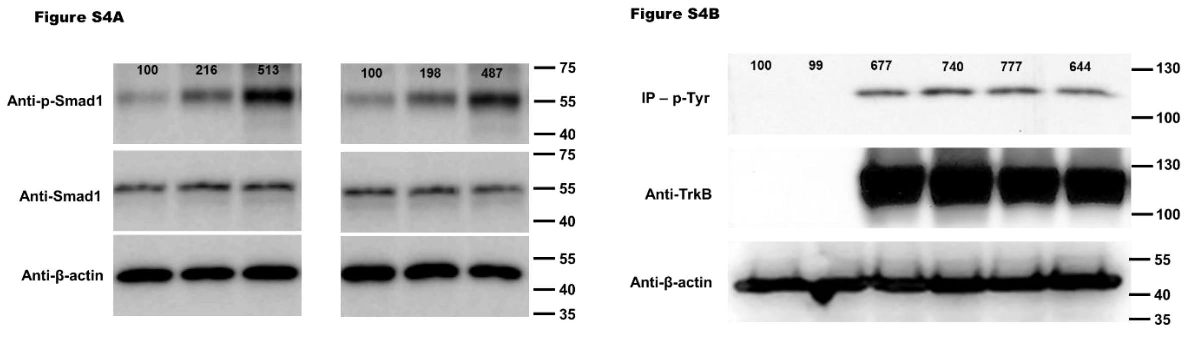

Figure S15. Detailed information about western blot of Figure S4A-B.

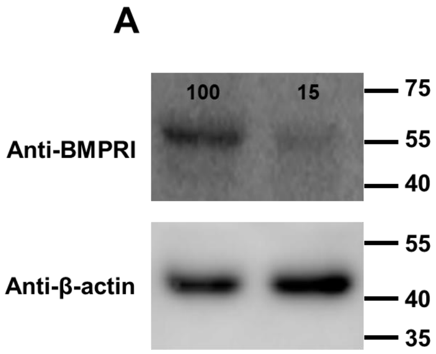

Figure S16. Detailed information about western blot of Figure S5A.

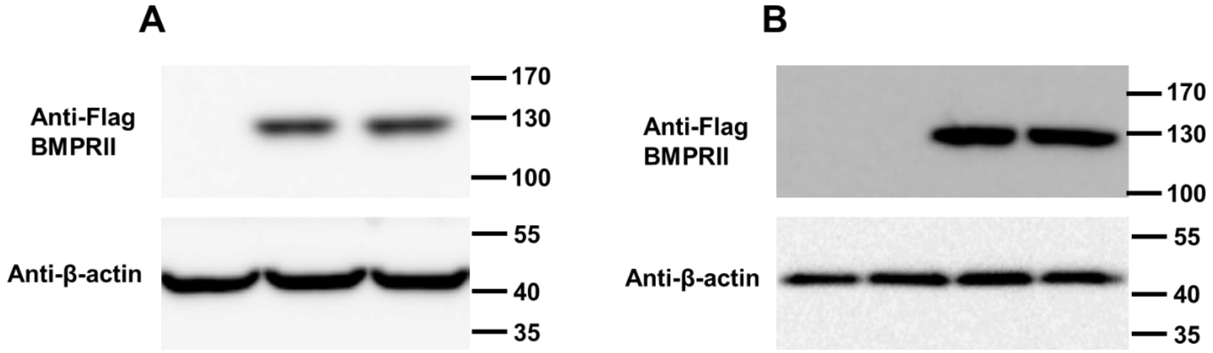

Figure S17. Detailed information about western blot of Figure S6A-B.
